# The Child Affective Facial Expression (CAFE) set: validity and reliability from untrained adults

**DOI:** 10.3389/fpsyg.2014.01532

**Published:** 2015-01-06

**Authors:** Vanessa LoBue, Cat Thrasher

**Affiliations:** Department of Psychology, Rutgers UniversityNewark, NJ, USA

**Keywords:** faces, emotions, expressions, CAFE, facial expressions

## Abstract

Emotional development is one of the largest and most productive areas of psychological research. For decades, researchers have been fascinated by how humans respond to, detect, and interpret emotional facial expressions. Much of the research in this area has relied on controlled stimulus sets of adults posing various facial expressions. Here we introduce a new stimulus set of emotional facial expressions into the domain of research on emotional development—The Child Affective Facial Expression set (CAFE). The CAFE set features photographs of a racially and ethnically diverse group of 2- to 8-year-old children posing for six emotional facial expressions—angry, fearful, sad, happy, surprised, and disgusted—and a neutral face. In the current work, we describe the set and report validity and reliability data on the set from 100 untrained adult participants.

Faces are a very special category of stimuli for us as humans. We might see hundreds of faces in the course of a day and millions in the course of a lifetime. Because of their importance to our everyday social interactions, perception of human facial expressions has likewise been an important topic in psychological research. Nearly three decades ago, Paul Ekman and colleagues identified six “basic” emotional expressions and argued that these six emotions are universally recognizable in most human populations. These emotions include sadness, happiness, surprise, anger, disgust, and fear (Ekman and Friesen, 1976; Ekman, 1992). Based on this early work, Ekman and Friesen (1976) created a stimulus set of photographs of adults posing these six basic expressions to facilitate research in this domain. The use of photograph stimulus sets of emotional facial expressions has since become standard practice, as they provide an easy and controlled way of examining human's interpretation and reaction to the various emotions. In fact, there are currently dozens of different face sets freely available for use in scientific research (www.face-rec.org/databases).

Despite their usefulness, facial expression sets are currently limited as most of them only capture the emotional expressions in adults. Very recently, researchers have begun to assert the importance of having child exemplars of the various emotional expressions represented in face sets in order to study the interpretation of these expressions developmentally. For example the new NIMH Child Emotional Faces Picture Set (NIMH-ChEFS) features photos of children aged 10–17 (Egger et al., 2011) and the Raboud Faces Database includes photos of 8- to 12-year-olds (Langner et al., 2010). Although these new sets give researchers the option of using exemplars of children aged 8–17, to date there are no stimulus sets featuring younger children.

Here we introduce a new and highly innovative stimulus set of emotional facial expressions to the domain of research in emotional development—The Child Affective Facial Expression Set (CAFE). The CAFE set is unique and useful for several important reasons. First and most important, the set features photographs of 2- to 8-year-old children posing the six basic emotions defined by Ekman—sadness, happiness, surprise, anger, disgust, and fear—plus a seventh neutral expression. It is also racially and ethnically diverse, featuring Caucasian, African American, Asian, Latino (Hispanic), and South Asian (Indian/Bangladeshi/Pakistani) children.

Although Ekman and others argue for a set of discrete or “basic” emotions that are purportedly universal and highly iconic, others have criticized this view, pointing out that people show wide individual differences in both emotional expression and emotional responsiveness (Barrett, 2006; Coan, 2010). The photographs in the CAFE contain a large amount of variability between faces to allow independent researchers to identify and study the natural variation in human facial expressions—variation that is not as easy to study in other smaller face sets. Thus, although the CAFE set only includes seven putatively basic emotions, the natural variation in the set will allow researchers to identify faces that are reminiscent of more subtle forms, or faces that are blends of multiple emotional expressions (Keltner and Buswell, 1996; Coan and Gottman, 2007). To allow for such variation, the CAFE set contains 1192 targets. Most other stimulus sets of adults' emotional facial expressions contain approximately 100 or fewer stimuli.

Finally, since there are 1192 photographs in the entire CAFE set, we not only offer the CAFE set in its entirety, but we have also identified two subsets of faces that researchers can choose from based on their specific research questions. Although previous researchers have used various methods to ensure the validity and reliability of the photographs in their face sets, very few have taken measures to ensure that the photographs do not produce ceiling or floor effects due to the fact that they are posed and often highly stereotypical exemplars of each emotion. CAFE includes one subset of faces (Subset A) that contains only highly stereotypical exemplars of the various facial expressions, consistent with other existing face sets, and a second subset (Subset B) that in contrast only includes faces that emphasize variation around emotion targets in research participants while minimizing potential ceiling and floor effects (as identified by latent response models).

In the current research, our goal was to collect validity and reliability data on the full set, and use these data to create its two subsets. We asked 100 naïve adult participants to identify the emotion posed in each photograph on two occasions. We report both validity and reliability statistics for each emotion category, and describe latent response models that we used to identify a subset of faces that will maximize variability in a typical sample of adult participants.

## Methods

The primary purpose of the current work was to obtain validity and reliability scores for each of the faces in the CAFE set so it can be disseminated to the scientific community. There are two ways that facial expressions are generally validated in the literature. One is to have trained coders use Ekman and colleagues' formal coding procedure—the Facial Affective Coding System (FACS)—to identify each facial expression (Ekman and Friesen, 1976). A second approach is to have untrained research participants identify each facial expression, and to then establish concordance between the raters (Tottenham et al., 2009). The advantage of using the formal FACS coding method is that it establishes uniformity among the various facial expressions. The advantage of the naïve coding approach is that it obtains the scores of participants who are similar to those who will be presented with these faces in standard research studies, typically undergraduates (Tottenham et al., 2009).

The current research employed a combination of these two methods. First, one of the authors (C.T.), trained in recognizing the specific muscle movements outlined by Ekman and colleagues, photographed all of the child models posing for the photographs in the CAFE set. Second, untrained participants were asked to identify each of the photographs in the set on two occasions. Thus, although a researcher highly trained in Ekman's facial coding system photographed the children, untrained participants were asked to identify the child expressions.

### Participants

One hundred undergraduate students (half male, half female) from the Rutgers University-Newark campus participated (*M* = 21.2 years). The sample size was based on previous research using similar methods (e.g., Tottenham et al., 2009). Data were collected from 17 additional adults but were excluded for failure to complete one of the two test sessions. The sample was 17% African American, 27% Asian, 30% White, and 17% Latino (the remaining 9% chose “Other” or did not indicate their race/ethnicity). The Rutgers University Institutional Review Board approved all procedures, and all participants signed an informed consent.

### Materials

The CAFE is a collection of photographs taken of 2- to 8-year-old children (*M* = 5.3 years; *R* = 2.7–8.7 years) posing for six emotional facial expressions based on Ekman and Friesen's (1976) basic emotional expressions—sadness, happiness, surprise, anger, disgust, and fear—plus a neutral face. The full set features 90 female models and 64 male models (27 African American, 16 Asian, 77 Caucasian/European American, 23 Latino, and 11 South Asian). With the exception of surprise, children were verbally prompted to pose for each expression with their mouths open and with their mouths closed. Surprised faces were only posed with their mouths open. Open mouth disgusted faces included a tongue protrusion. In total, we had 154 child-models (90F, 64M) pose each of these seven expressions. The children were photographed in a lab setting after attaining permission from their parent or guardian. The children were all visiting the lab to participate in another study, and parents agreed to allow their children to be photographed for the CAFE set afterward. Parents of the participating children signed a model release giving permission for the use of their photographs in research by the greater scientific community. Child models had no prior training. A professional photographer (co-author C.T.) elicited naturalistic expressions by engaging each child in unscripted play based on each expression. All of the photos were taken against the same off-white background with overhead lighting. In addition, each child was covered from the neck down with an off-white sheet.

**Figure 1:**
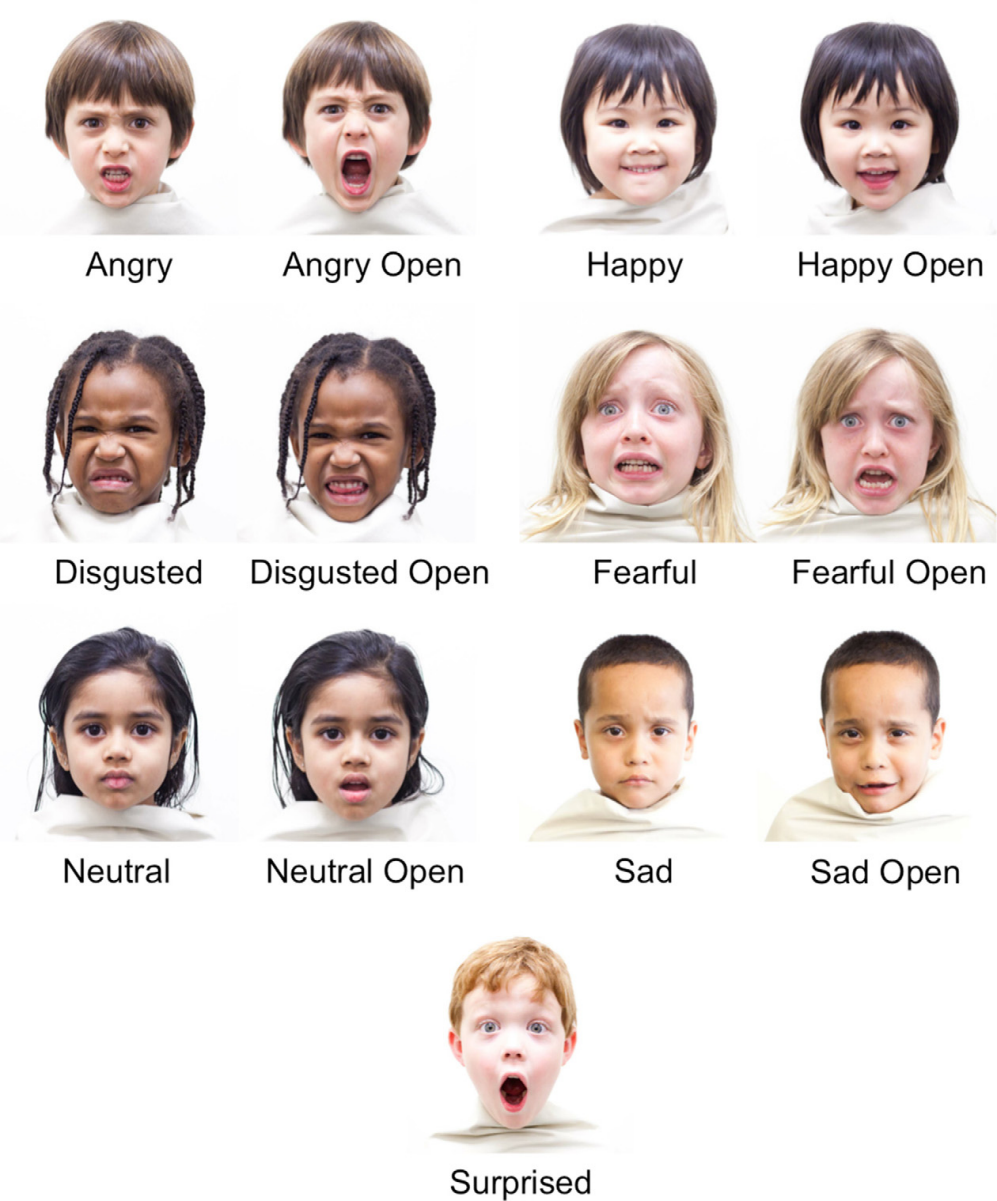
**Examples of each of the posed facial expressions in the CAFE set**.

The photographer was a trained research assistant with several years of experience working in a child development lab. Most importantly, the photographer was trained in the Specific Affect (SPAFF) coding system (Coan and Gottman, 2007). SPAFF is a systematic coding system used to evaluate affective behaviors. The SPAFF system includes procedures for recognizing facial muscle movements associated with 17 codable emotional states in real time, incorporating elements of the FACS coding system designed by Ekman and colleagues (Ekman and Friesen, 1976). Thus, for each facial expression, the photographer was able to recognize the presence or absence of FACS codes during the photo session, and for each child and each facial expression, she attempted to obtain all of the elements of the FACS codes (e.g., angry faces include brows down, upper eye lids raised, clenched jaw, teeth showing). The child-models were given various instructions. First, the photographer instructed the child to make each facial expression by modeling it. For example, “… now we're going to make an angry face, like this!” After the child imitated the initial facial expression, the photographer assessed whether there were missing elements of the facial expression based on the FACS codes. If certain facial elements were missing, the photographer prompted the children to revise their facial expressions. For example, “… show me those teeth!” or “… just like that, except let me see those eyes get big like this!” Not all children were able to successfully pose for all seven expressions, and thus, all unsuccessful attempts were eliminated from the set. The result was 1192 total color photographs (see Figure 1).

The 1192 facial expressions included (1) several “target” expressions associated with a small set of seven putatively “basic” emotions; and (2) a wide range of variation in expression around those targets. In this way, the stimulus set is flexible enough to provide examples of what may now be considered classic “Ekman” expressions in addition to a large variety of more nuanced, variable, and subtle expressions representing deviations by degree (some are quite similar, some quite different) from those “Ekman” expressions. Once the photographs were taken, they were cropped to a square image, with the child's chin approximately 1/6 from the bottom of the image (based on the image's height), and the child's forehead approximately 1/6 from the top. They were aligned based on the individual child's eye alignment, using the points on the outside of each eye as a reference.

### Procedure

Each of the 100 adult participants sat in front of an Ibuypower I-Series 502 desktop computer with a 22″ screen. They were presented with 1192 trials on *E-prime*, each of which required them to identify one of the photos from the CAFE set. On each successive trial, one face appeared on the screen. For each face, the participant was prompted to choose whether the face was sad, happy, surprised, angry, disgusted, fearful, or neutral. The photographs were presented in a random order. Participants completed all 1192 trials in a single test session (Time 1), and then returned 1 week later and completed all 1192 trials for a second time (Time 2). Each test session lasted approximately an hour and a half.

## Results

### Item-level data

The photographs in the set and the item level data described below are available for download on Databrary—a free, open data library for developmental science (www.databrary.org). Faculty members can register with Databrary to become users and gain access to the photographs in the set and to a sortable Excel file with demographic information and the data on each individual item. Once registered, faculty can grant graduate students, post-docs, and staff permission to access the set as well. Visit www.databrary.org for more information about becoming a Databrary user; the set itself can be accessed directly by users here http://databrary.org/volume/30.

### Full cafe set

**Figure 2:**
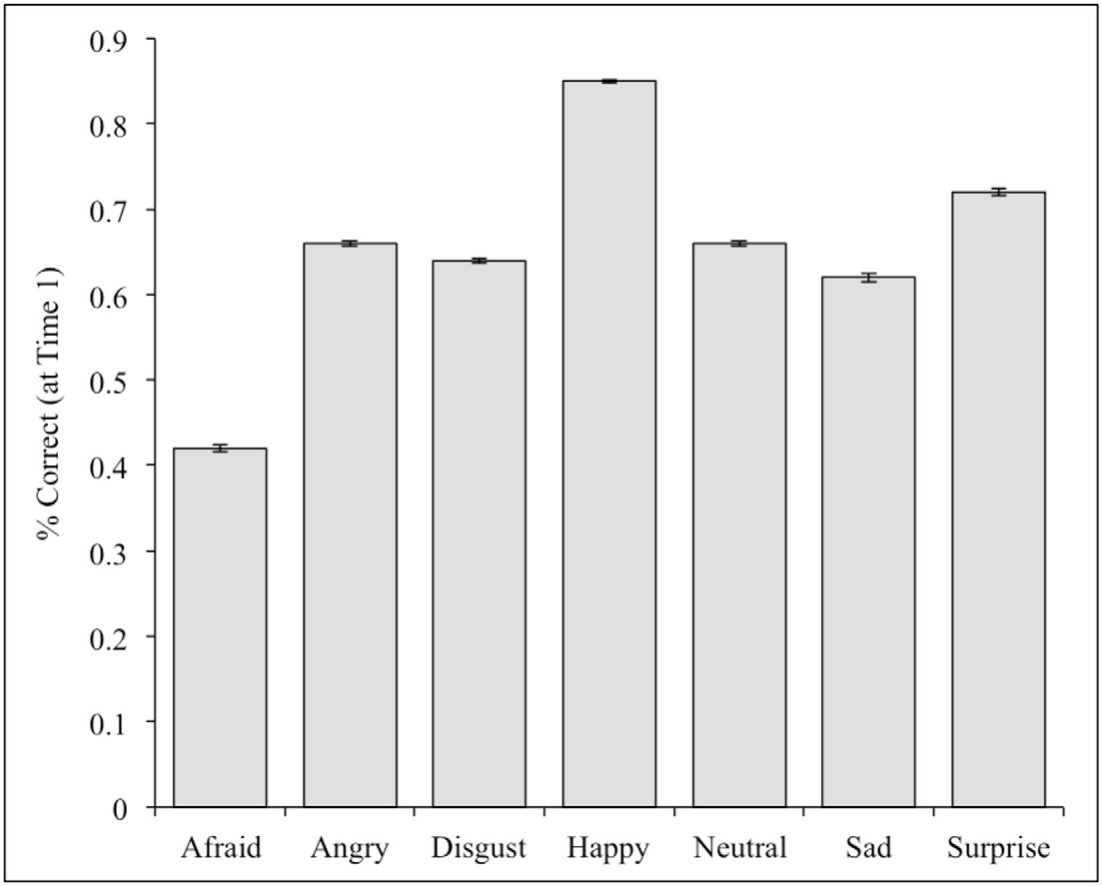
**Percent accuracy for each category of facial expression in the full CAFE set at Time 1**.

Validity scores were calculated by obtaining the percentage of the 100 participants to correctly categorize the photographs at Time 1. The percent correct for each individual face is provided in the supplementary materials, and the means for each of the seven expressions are listed in Tables 1, 2 (open mouth and closed mouth separately). There was substantial variability across the faces, with a mean of 66% accuracy across the 1192 photographs of the set, and a range of 0–98% correct. We measured internal consistency (reliability) by calculating Cronbach's alpha scores between Time 1 and Time 2. The alpha was high on the overall set, α = 0.77. Alpha scores for each emotion category are also listed in Tables 1, 2. There were significant differences in accuracy for the seven categories of facial expressions according to a One-Way ANOVA, *F*_(1, 6)_ = 262.7, *p* < 0.01. *Post-hoc* comparisons (*Tukey-d)* indicated that the categories were all significantly different from each other (*p's* < 0.01) with the exceptions of angry vs. neutral (*p* = 0.99), and sad vs. disgust (*p* = 0.061) (see Figure 2).

**Table 1:** **Descriptive statistics for seven categories of facial expressions in the full CAFE Set**.

**Emotion**	***N***	**Mean % correct (Time 1)**	**Mean % correct (Time 2)**	**Std deviation (Time 1)**.	**Std. error of mean (Time 1)**	**Cronbach's alpha (T1,T2)**
Afraid	140	0.42	0.49	0.50	0.004	0.46
Angry	205	0.66	0.65	0.47	0.003	0.50
Disgust	191	0.64	0.66	0.48	0.003	0.50
Happy	215	0.85	0.83	0.36	0.002	0.40
Neutral	230	0.66	0.65	0.48	0.003	0.50
Sad	108	0.62	0.63	0.49	0.005	0.52
Surprise	103	0.72	0.65	0.45	0.004	0.42
Total	1192	0.66	0.66	0.47	0.001	0.77

**Table 2:** **Descriptive statistics for seven categories of facial expressions in the full CAFE Set with mouth open and closed expressions listed separately**.

**Emotion**	***N***	**Mean % correct (Time 1)**	**Mean % correct (Time 2)**	**Std deviation (Time 1)**	**Std. error of mean (Time 1)**	**Cronbach's alpha (T1,T2)**
Afraid	79	0.45	0.52	0.50	0.006	0.42
Afraid open	61	0.38	0.46	0.49	0.006	0.44
Angry	121	0.66	0.64	0.48	0.004	0.52
Angry open	84	0.66	0.68	0.47	0.005	0.47
Disgust	96	0.54	0.56	0.50	0.005	0.55
Disgust open	95	0.73	0.77	0.44	0.005	0.37
Happy	120	0.93	0.91	0.25	0.002	0.16
Happy open	95	0.74	0.73	0.44	0.005	0.40
Neutral	129	0.86	0.84	0.35	0.003	0.37
Neutral open	101	0.40	0.40	0.49	0.005	0.47
Sad	62	0.75	0.74	0.43	0.005	0.48
Sad open	46	0.45	0.47	0.50	0.007	0.48
Surprise	103	0.72	0.65	0.45	0.004	0.42
Total	1192	0.66	0.66	0.47	0.001	0.77

### Subset A

The full CAFE set has a large number of photographs and participants' overall accuracy in identifying each face was low (66%), reflecting a high degree of variability in their accuracy scores. Thus, a selection of the faces will be offered as a separate subset, containing only the photographs that were identified with 60% accuracy or more at Time 1. Based on this restriction, Subset A was created containing 789 of the original 1192 (66%) photographs. Descriptive statistics for each of the seven categories of facial expressions are listed in Tables 3, 4 (open mouth and closed mouth separately). The overall accuracy in Subset A was 81%, higher than in the full set, as was the alpha score, α = 0.82. Again, there were significant differences in accuracy among the seven categories of facial expressions according to a One-Way ANOVA on Subset A, *F*_(1, 6)_ = 266.0, *p* < 0.01. *Post-hoc* comparisons (*Tukey-d)* indicated that the emotions were all significantly different from each other (*p's* < 0.01) with the exceptions of angry vs. sad (*p* = 0.68), and surprise vs. disgust (*p* = 0.75).

**Table 3:** **Descriptive statistics for seven categories of facial expressions in Subset A of the CAFE Set**.

**Emotion**	***N***	**Mean % correct (Time 1)**	**Mean % correct (Time 2)**	**Std deviation (Time 1)**	**Std. error of mean (Time 1)**	**Cronbach's alpha (T1,T2)**
Afraid	25	0.70	0.72	0.46	0.009	0.32
Angry	141	0.80	0.78	0.40	0.003	0.35
Disgust	127	0.75	0.76	0.43	0.004	0.35
Happy	194	0.89	0.87	0.32	0.002	0.29
Neutral	153	0.84	0.82	0.37	0.003	0.36
Sad	65	0.79	0.77	0.41	0.005	0.43
Surprise	84	0.76	0.68	0.43	0.005	0.41
Total	789	0.81	0.79	0.39	0.001	0.82

**Table 4:** **Descriptive statistics for seven categories of facial expressions in Subset A with mouth open and closed expressions listed separately**.

**Emotion**	***N***	**Mean % correct (Time 1)**	**Mean % correct (Time 2)**	**Std deviation (Time 1)**	**Std. error of mean (Time 1)**	**Cronbach's alpha (T1,T2)**
Afraid	20	0.70	0.73	0.46	0.010	0.33
Afraid open	5	0.69	0.70	0.46	0.021	0.28
Angry	83	0.81	0.78	0.40	0.004	0.32
Angry open	58	0.78	0.79	0.41	0.005	0.36
Disgust	42	0.73	0.72	0.45	0.007	0.37
Disgust open	85	0.76	0.79	0.43	0.005	0.33
Happy	120	0.93	0.91	0.25	0.002	0.16
Happy open	74	0.82	0.81	0.39	0.004	0.30
Neutral	126	0.86	0.85	0.34	0.003	0.36
Neutral open	27	0.71	0.67	0.45	0.009	0.37
Sad	49	0.82	0.80	0.39	0.006	0.41
Sad open	16	0.69	0.69	0.46	0.012	0.42
Surprise	84	0.76	0.68	0.43	0.005	0.41
Total	789	0.81	0.79	0.39	0.001	0.82

### Subset B

In order to identify a subset of faces that permits substantial variability while minimizing floor and ceiling effects, we applied a one-parameter logistic or *Rasch model* to assess the difficulty of identifying photographs within each emotion set based on the obtained participant identifications at Time 1. The model was based on the following equation:

$$P_{i}(\theta )=\frac{e[\theta -b_{i}]}{1+e[\theta -b_{i}]}$$

In the equation, θ represents a participant's true ability to correctly identify each expression, *P_i_(θ)* represents the probability of a random participant correctly identifying expression *i*, and *b_i_*represents the probability of correctly identifying expression *i* at *P_i_(θ)* = 0.5 or 50% (Linacre and Wright, 1994).

For Subset B, we used this model to calculate a difficulty score (*b_i_*), along with fit statistics (in-fit and out-fit), for each photograph in order to select a subset of faces that varied substantially within emotion category, but also could still be said to represent each category. First, we calculated a standardized difficulty score for each face (*b_i_*). Participants' ability to correctly identify each face was standardized on a continuum, such that difficulty scores for each item (*b_i_*) ranged from positive to negative, with more positive scores indicating greater difficulty, and more negative scores indicating lower difficulty. Here, *difficulty* refers to the level of ability required to correctly identify an expression in the image provided. When scores are more negative, then, most individuals can correctly identify the expression in that image, most of the time, because these expressions are “easy” in the sense that relatively low levels of ability in identifying expressions of the type in question (happy, fear, etc..) are required in order to correctly identify them. By contrast, more positive scores indicate that relatively few individuals will correctly identify the expression in the image provided, most of the time, because higher levels of ability in identifying expressions of the type in question (happy, fear, etc…) are required in order to correctly identify them.

**Figure 3:**
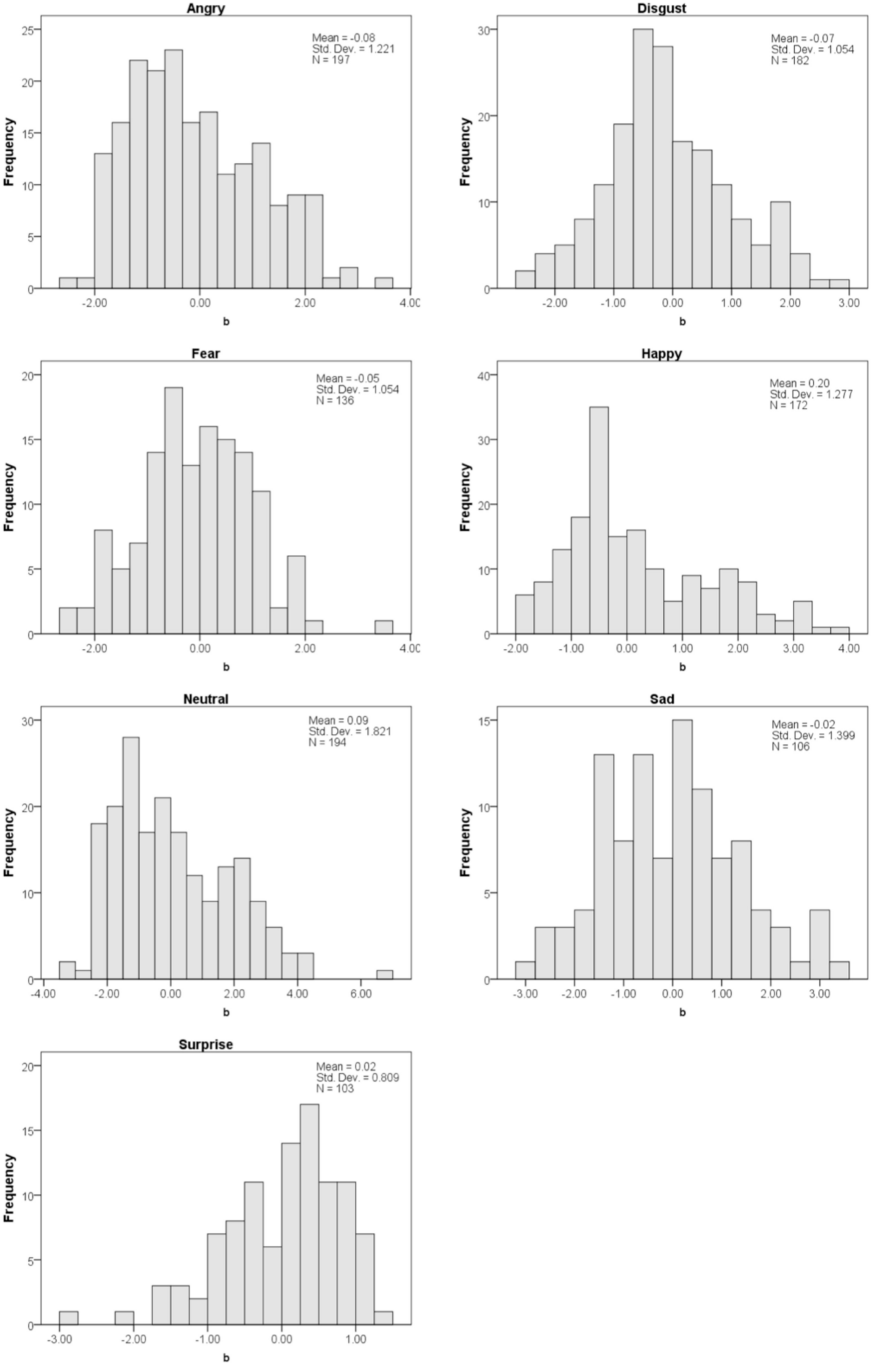
**Distribution of difficulty (*b_i_*) scores for each emotion category**.

Next, we used the in-fit and out-fit mean square statistics to narrow down the faces in the Subset. The in-fit is an index of unexpected responses to items that have a difficulty score (*b_i_*) that is close to an individual's ability (e.g., cases where an individual responds incorrectly to an item that is easy with respect to his/her ability). The out-fit is an index of unexpected responses to items that have a difficulty score (*b_i_*) that is far from an individual's ability (e.g., cases where an individual responds correctly to an item that is too difficult for his/her ability). In-fit and out-fit scores lower than 0.5 indicate a lack of reliability, whereas in-fit and out-fit scores greater than 1.5 indicate noise (Linacre and Wright, 1994). Thus, Subset B is comprised of the faces that fit within the 0.5–1.5 range. 102 of the 1192 faces fell outside of this range, leaving Subset B with 1090 total photographs. The difficulty scores (*b_i_*) for each of the seven emotional categories are plotted in histograms in Figure 3, demonstrating that the mean difficulty for each category is close to zero, and that the distribution of scores for each emotional expression is fairly normal. A One-Way ANOVA on the difficulty scores between the emotional facial expressions was not statistically significant, *F*_(1, 1083)_ = 1.03, *p* = 0.41, confirming that the mean difficulty for each category of facial expression is similar (and close to zero).

Descriptive statistics for each of the seven types of facial expressions in Subset B are listed in Tables 5, 6 (open mouth and closed mouth separately). The overall accuracy was the same as in the full set (66%), as was the alpha, α = 0.768. Again, there were significant differences in accuracy among the seven categories of facial expressions according to a One-Way ANOVA on Subset B, *F*_(1, 6)_ = 974.0, *p* < 0.01. *Post-hoc* comparisons (*Tukey-d)* indicated that the emotion categories were all significantly different from each other (*p's* < 0.01) with the exception of disgust vs. neutral (*p* = 1.000).

**Table 5:** **Descriptive statistics for seven categories of facial expressions in Subset B of the CAFE Set**.

**Emotion**	***N***	**Mean % correct (Time 1)**	**Mean % correct (Time 2)**	**Std deviation (Time 1)**	**Std. error of mean (Time 1)**	**Cronbach's alpha (T1,T2)**
Afraid	136	0.43	0.50	0.50	0.004	0.46
Angry	197	0.67	0.67	0.47	0.003	0.48
Disgust	182	0.65	0.67	0.48	0.004	0.46
Happy	172	0.83	0.82	0.38	0.003	0.41
Neutral	194	0.65	0.64	0.48	0.003	0.49
Sad	106	0.62	0.63	0.48	0.005	0.51
Surprise	103	0.72	0.65	0.45	0.004	0.42
Total	1090	0.66	0.66	0.47	0.001	0.77

**Table 6:** **Descriptive statistics for seven categories of facial expressions in Subset B with mouth open and closed expressions listed separately**.

**Emotion**	***N***	**Mean % correct (Time 1)**	**Mean % correct (Time 2)**	**Std deviation (Time 1)**	**Std. error of mean (Time 1)**	**Cronbach's alpha (T1,T2)**
Afraid	77	0.46	0.53	0.50	0.006	0.42
Afraid open	59	0.39	0.46	0.49	0.006	0.44
Angry	116	0.68	0.66	0.47	0.004	0.48
Angry open	81	0.66	0.68	0.47	0.005	0.47
Disgust	88	0.56	0.57	0.50	0.005	0.51
Disgust open	94	0.73	0.77	0.44	0.005	0.37
Happy	86	0.93	0.91	0.26	0.003	0.13
Happy open	86	0.73	0.73	0.45	0.005	0.41
Neutral	105	0.85	0.84	0.36	0.003	0.37
Neutral open	89	0.41	0.40	0.49	0.005	0.46
Sad	61	0.75	0.74	0.43	0.006	0.48
Sad open	45	0.46	0.48	0.50	0.007	0.47
Surprise	103	0.72	0.65	0.45	0.004	0.42
Total	1090	0.66	0.66	0.47	0.001	0.77

### Group differences

We analyzed group differences to examine whether gender and race/ethnicity of the adult participants and of the child models affected accuracy in correctly identifying emotional expressions. First we examined characteristics of the adult participants. Participants self-identified as Caucasian, African American, Asian, Latino, or Other. Preliminary analyses indicated that there were no significant interactions between participant's gender/race/ethnicity and emotion category (e.g., angry, sadness, etc..) so emotion category was not included in further analyses. A 2 (participant gender: male, female) by 5 (participant race/ethnicity: Caucasian, African American, Asian, Latino, or Other) ANOVA on average proportion of correct responses to the faces in each of the seven categories yielded only a significant main effect of gender, *F*_(1, 690)_ = 7.0, *p* = 0.008. Female adults were significantly more accurate (*m* = 0.68) than were males (*m* = 0.63) at identifying all expressions.

A similar analysis was done on the characteristics of the child models, including gender (male vs. female), race/ethnicity (Caucasian, African American, Asian/South Asian, Latino, or Other), and age (2–5.5 years vs. 5.5–8 years). In a 2 (model's gender) by 5 (model's race/ethnicity) by 2 (model's age) ANOVA on average proportion of correct responses to the faces in each of the seven categories, there were no significant main effects or interactions. Additional analyses were done breaking down age of the child models by year (2-, 3-, 4-, 5-, 6-, 7-, and 8-year-olds) and there were again no significant effects.

Finally, we performed two additional analyses to examine whether participants were more accurate at identifying faces that matched their own gender and race/ethnicity than faces of the opposite gender or other races/ethnicities. A 2 (match vs. mismatch race/ethnicity) by 5 (participant's race/ethnicity: Caucasian, African American, Asian, Latino, or Other) ANOVA on average proportion of correct responses to each of the seven categories of facial expressions yielded no significant differences or interactions. A second 2 (match vs. mismatch gender) by 2 (participant gender: male vs. female) yielded only a significant main effect of participant gender (the same main effect as in the analysis above), but no effect of whether participants were more accurate when the gender of the model matched their own.

## General discussion

Validated stimulus sets of photographed emotional facial expressions are commonly used in social, cognitive, and developmental research. However, until recently, these stimulus sets mainly featured adult faces, limiting our ability to study the interpretation of emotions at different ages. Here we introduce a new stimulus set to the field of emotion research—The CAFE. CAFE features photographs of a group of ethnically and racially diverse 2- to 8-year-old children posing six emotional expressions plus a neutral face. Data on the validity and reliability of the face set from 100 untrained adults suggests that it is a viable tool for studying emotional expressions in the research community. Further, we have used data from the validation of the set to create two subsets that will further aid researchers in choosing faces that best fit their individual research needs.

The full set is most useful if researchers are interested in a particular demographic, or in choosing a select group of faces that fit with particular criteria. For example, if researchers are interested in using faces from a particular race or ethnicity, it would be most useful to select from the full set in order to maximize the number of useable exemplars. Alternatively, if researchers are interested in using faces that are ambiguous, or they are interested in emotional or negative blends, they can do so by carefully selecting from faces in the full set. For example, the target emotion for item Angry_F-AA-08 is angry, but while 31% of participants correctly identified the face as angry, 33% identified it as disgusted, and 26% identified it as fearful, suggesting that the negative emotion portrayed by the face is quite ambiguous. Since the set is so large (with 1192 exemplars), researchers have the flexibility to choose faces that meet very specific criteria, and the full set is ideal for this purpose.

For researchers who are not interested in ambiguity, and instead require faces that depict iconic and easily recognizable exemplars of various emotional expressions, Subset A provides that option, and only contains faces that 60% of participants or more identified correctly. This subset of faces is similar to previous face sets that are highly recognizable. Despite its narrower scope compared to the full CAFE set, Subset A still has 789 items, making it feasible for researchers to further narrow their selection of faces within the subset based on specific criteria (e.g., African American happy faces; sad 4-year-old faces).

Finally, researchers interested in variability around target expressions can choose to use Subset B. The faces in Subset B are reliable, but vary on a normal distribution in terms of how difficult they are to identify. Again, difficulty refers here to the putative level of ability required to correctly identify an expression from the image provided. Only some of the items are iconic and easy to classify, some of the items are difficult, and most lie somewhere in the middle. Unreliable or noisy items have been eliminated from this subset, meaning in part that “difficult” items were not identified by high levels of accuracy, nor simply by their relative infrequency, but rather by the degree to which only participants with the highest individual ability levels (as assessed by our Rasch-model) were able to correctly identify them. In this way, Subset B can be used as stimuli or, potentially, as a diagnostic tool, since the faces in this subset are capable of providing information on an individual's ability to recognize human emotions.

For future research it will be important to collect data on children's identifications of the CAFE faces in order to best assess which faces are identified the most accurately at various ages. Previous work on the “own-age bias,” has already demonstrated that children recognize faces the most accurately when they are within 2 years of their own age (Hills and Lewis, 2011). Until we have collected additional data on children's identification of the faces in the CAFE set, researchers can use this previous work on the own-age bias as a rule of thumb for when the faces might be most appropriately used with children of various ages. Relatedly, future work can also compare both adults' and children's identifications of the child faces in the CAFE set to identifications of adult faces in other sets. Such data would provide interesting information about whether there are developmental differences in children and adults' abilities to both pose and identify the various emotional facial expressions.

In conclusion, here we present a new stimulus set to investigators interested in the study of emotional facial expressions. The already frequent use of facial expression sets in psychological research suggests that this set has the potential of making a large and important impact on the field. It will allow the scientific community to conduct research with children's faces in a manner that is comparable to a large extant literature that has heavily relied on adult faces. Thus, the CAFE set breaks new ground by using a diverse set of naturalistic child facial expressions, while maintaining a medium that makes it comparable to an already large literature.

### Conflict of interest statement

The authors declare that the research was conducted in the absence of any commercial or financial relationships that could be construed as a potential conflict of interest.
